# Sex under the moon

**DOI:** 10.7554/eLife.12936

**Published:** 2015-12-15

**Authors:** Didier Zoccola, Sylvie Tambutté

**Affiliations:** Marine Biology Department, Centre Scientifique de Monaco, Monaco, Europezoccola@centrescientifique.mc; Marine Biology Department, Centre Scientifique de Monaco, Monaco, Europe

**Keywords:** coral, cnidarians, mass spawning, reproduction, signalling cascade, moon light, Other

## Abstract

Moonlight alters the expression of a number of genes in coral cells in order to synchronize the release of sex cells across different coral species.

**Related research article** Kaniewska P, Alon S, Karako-Lampert S, Hoegh-Guldberg O, Levy O. 2015. Signaling cascades and the importance of moonlight in coral broadcast mass spawning.*eLife* **4**:e09991. doi: 10.7554/eLife.09991

**Image** An *Acropora millepora* colony during a mass spawning event on the Great Barrier Reef.

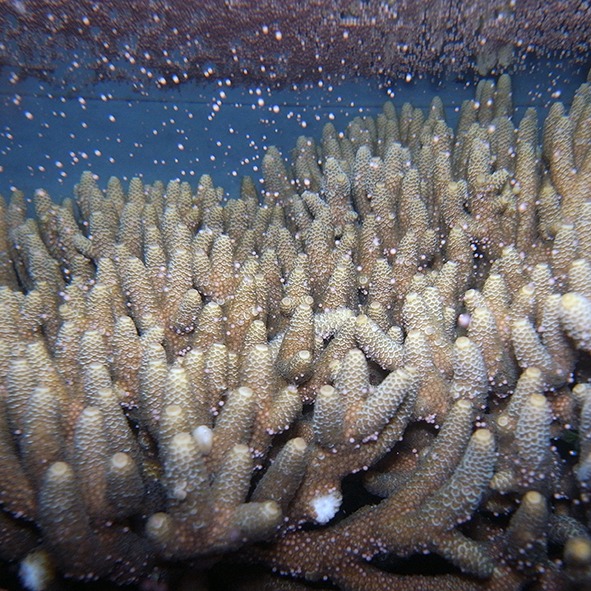

The moon has always fascinated humans. Lunar cycles have dominated human culture for thousands of years, with the first lunar calendars generated by early civilizations. Although folklore and myths have long linked some aspects of human behavior to the lunar cycle (as seen in the many tales featuring werewolves), the actual effect of the moon on human physiology is still debated (reviewed by Foster and Roenneberg, 2008). There is, however, clear evidence that the behavior of many animals is influenced by the moon. This is especially true for reef-building corals, where the process of spawning depends on lunar cycles (Harrison, 2011).

Ever since the 1930s, when Sheina Marshall and Alan Stephenson published their report on an expedition to the Great Barrier Reef (Marshall and Stephenson, 1933), scientists have been puzzled by the processes of breeding in corals. Most corals spawn by releasing gametes – egg and sperm cells – into the sea, where they combine and develop into larvae. To improve their chances of successfully reproducing, different corals often spawn simultaneously: a process known as mass spawning. On some parts of the Great Barrier Reef, for example, 20–30 coral species have been recorded spawning at the same time in the late spring and early summer, immediately after the full moon (Harrison et al., 1984). However, we do not fully understand how external factors, such as moonlight, affect the molecular mechanisms inside the coral cells so that spawning can be harmonized.

Now, in eLife, Oren Levy at Bar-Ilan University and co-workers – including Paulina Kaniewska and Shahar Alon as joint first authors – address this gap in our knowledge by studying gene expression during spawning in *Acropora millepora*, one of the coral species that makes up the Great Barrier Reef (Kaniewska et al., 2015). A combination of field and outdoor aquaria observations revealed that the pattern of light exposure experienced by corals is fundamental for the synchronization of spawning. Indeed, of the colonies maintained in aquaria, only those exposed to natural conditions of daylight and moonlight spawned in a similar manner to those in the reef. Furthermore, the expression of genes linked to rhythmic processes and light sensing such as cryptochromes varied relative to the level of moonlight the corals were exposed to.

But how do corals detect light? From a histological and anatomical point of view, corals are very simple animals that do not possess specialized visual structures. However, they do contain light-sensitive molecules such as opsins and cryptochromes (Reitzel et al., 2013). Now, Kaniewska et al. – who are based at the Australian Institute of Marine Science, the University of Queensland, Tel Aviv University and Bar-Ilan University – suggest that gamete release in corals is triggered by a protein similar to a photosensitive molecule called melanopsin.

Melanopsin was first discovered in photosensitive pigment granules in the skin of frogs that can change color in response to light (Provencio et al., 1998): this change of color is achieved by reorganizing the pigment granules within cells. In mammals it has been shown that melanopsin plays a critical role in synchronizing circadian rhythms with the daily light-dark cycle. Moreover, melanopsin is capable of catalyzing the activation of G-proteins – proteins that help transmit signals from outside a cell to the appropriate destination inside it – in response to changes in light conditions (Brown and Robinson, 2004). During moonlight, Kaniewska et al. observed the up-regulation of processes linked to a G-protein activation cascade in corals. This suggests that this pathway could be responsible for moonlight sensing. However, further research is needed to find out if the melanopsin-like proteins in corals are true photopigments.

Another factor that may be central to controlling the release of gametes is neuropeptide signaling. Corals have no brain but they possess a diffuse nervous system that secretes neuropeptides that act on particular receptor proteins. Kaniewska et al. found that changes in the expression of neuropeptide receptors – primarily members of the large family of G-protein coupled receptors – occur during coral spawning.

Melanopsin-like proteins, neuropeptides and G-protein-coupled receptors all act at the surface of coral cells. But what happens inside the cell to trigger spawning? Cell surface receptors often send signals via calcium ions, and the photo-responsive cells of corals detect and respond to light by altering the levels of calcium in their cytoplasm (Hilton et al., 2012). Activating G-protein coupled receptors induces an increase in the amount of calcium inside cells. However, Kaniewska et al. show that ion channels belonging to the transient receptor potential superfamily are also upregulated during spawning, providing another route by which calcium ions can enter cells.

The properties of the moonlight also appear to influence the spawning behavior of the coral. Previous work has suggested that blue light detection is important for gamete release and for larvae settlement (Gorbunov and Falkowski, 2002). Kaniewska et al. provide further evidence for this theory by finding that specific parts of the light spectrum – particularly blue and green light – are key for gamete release. However, more work is needed to better understand the molecular basis of this selectivity.

In conclusion, this study lays the foundation for future studies on coral spawning, and demonstrates how molecular approaches can contribute to an improved understanding of the complex biological processes that take place in coral. However, molecular approaches must be complemented by investigations at the protein, cell and organism level to confirm the molecular data. Opportunities for future research into spawning include comparing how different coral species respond to moonlight and studying the effect of the light spectrum on gamete release.

There is also much to learn about coral species that reproduce on other lunar nights, during different lunar phases or at other times of the year. Furthermore, we can also learn more about coral species that release larvae called planulae and for which spawning is less synchronized.
